# Exosomal miRNAs versus circulating tumor DNA: diagnostic accuracy for early detection of recurrence post-radical prostatectomy: A narrative review

**DOI:** 10.1097/MD.0000000000046239

**Published:** 2025-11-28

**Authors:** Hamza Bashir, Muhammad Waqas Afzal, Asad Mehmood, Maham Afzal, Muhammad Wasi Hassan, Abida Parveen

**Affiliations:** a Department of Medicine, Ibn e Seena Hospital, Kabul, Afghanistan.

**Keywords:** biochemical recurrence, biomarker, circulating tumor DNA, early detection, exosomal microRNAs, liquid biopsy, minimal residual disease, prostate cancer, radical prostatectomy

## Abstract

Prostate cancer is a leading malignancy worldwide, and while radical prostatectomy is an effective treatment for localized disease, a significant proportion of patients experience biochemical recurrence. Prostate-specific antigen (PSA) remains the primary biomarker for recurrence detection, but its limitations in specificity necessitate improved surveillance tools. Liquid biopsy techniques, particularly exosomal microRNAs (miRNAs) and circulating tumor DNA (ctDNA), have emerged as promising noninvasive biomarkers for early recurrence detection. However, their comparative diagnostic accuracy remains unclear. This narrative review explores the diagnostic potential of exosomal miRNAs and ctDNA for early recurrence detection following radical prostatectomy. It examines their biological characteristics, detection methodologies, comparative diagnostic performance, and clinical challenges. Both exosomal miRNAs and ctDNA hold significant potential for the early detection of prostate cancer recurrence post-radical prostatectomy. However, the lack of standardized protocols and the variability in current studies limit definitive conclusions about their relative accuracy. Future large-scale, prospective studies are necessary to validate their clinical utility and establish guidelines for their integration into routine surveillance. A multi-biomarker approach combining exosomal miRNAs, ctDNA, and PSA may provide the most effective strategy for personalized prostate cancer monitoring.

## 1. Introduction

Prostate cancer is one of the most prevalent malignancies worldwide, and radical prostatectomy remains a cornerstone treatment for localized disease.^[1]^ However, biochemical recurrence (BCR) occurs in a significant proportion of patients, necessitating close post-surgical monitoring. The early and accurate detection of recurrence is crucial for timely intervention and improved clinical outcomes.^[2]^ Traditionally, prostate-specific antigen (PSA) levels serve as the primary biomarker for recurrence detection, but PSA alone lacks specificity and may not always reflect true disease progression.^[3]^ In recent years, liquid biopsy techniques – specifically exosomal microRNAs (miRNAs) and circulating tumor DNA (ctDNA) – have emerged as promising noninvasive biomarkers with potential for enhancing early recurrence detection.^[4]^ However, their relative diagnostic accuracy and clinical utility remain uncertain.

Exosomal miRNAs, encapsulated within extracellular vesicles, play critical roles in intercellular communication and are increasingly recognized as stable and specific biomarkers for cancer detection and progression monitoring.^[5]^ Meanwhile, ctDNA, shed by tumor cells into the bloodstream, offers insights into tumor genetics and clonal evolution.^[6]^ Both biomarkers hold promise for detecting minimal residual disease and early recurrence post-radical prostatectomy, but their comparative effectiveness has not been systematically evaluated. This systematic review aims to assess and compare the diagnostic accuracy of exosomal miRNAs and ctDNA in detecting prostate cancer recurrence after radical prostatectomy. By synthesizing existing evidence, this review will provide insights into the strengths and limitations of these biomarkers, guiding future research and clinical applications.

## 2. Methodology

A literature search was conducted using PubMed, Embase, Web of Science, and Scopus for English-language articles published up to June 2025. Search terms included “prostate cancer,” “radical prostatectomy,” “BCR,” “liquid biopsy,” “exosomal microRNA,” and “ctDNA.” Studies were included if they addressed the diagnostic role of exosomal miRNAs or ctDNA in detecting recurrence after radical prostatectomy. Articles focusing on unrelated biomarkers, non-human studies, or treatment efficacy were excluded. Relevant studies were selected, reviewed, and thematically synthesized to compare biological features, detection methods, diagnostic performance, and clinical applicability.

## 3. Discussion

### 3.1. Liquid biopsy in post-radical prostatectomy surveillance

The detection of prostate cancer recurrence following radical prostatectomy remains a major clinical challenge, as early intervention significantly impacts patient outcomes. While PSA remains the standard biomarker for post-surgical monitoring, its limitations in specificity often lead to uncertainty, necessitating more reliable biomarkers.^[7]^ Liquid biopsy techniques, particularly miRNAs and ctDNA, have gained attention as promising alternatives.^[8]^ These biomarkers provide a noninvasive means of detecting molecular changes associated with tumor recurrence, offering a more precise approach compared to PSA alone.^[9]^

**Figure 1.**
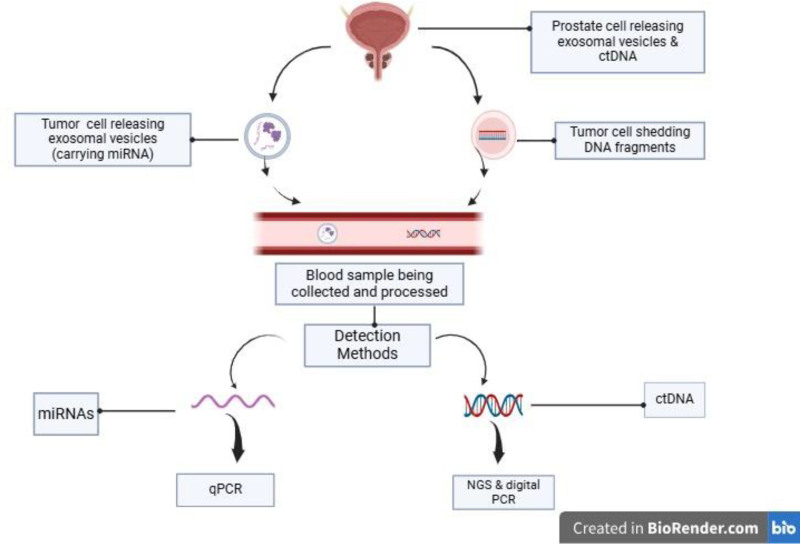
Overview of liquid biopsy biomarkers for prostate cancer recurrence.

Figure 1 illustrates how exosomal miRNAs and ctDNA are released by tumor cells into the bloodstream, where they can be detected through liquid biopsy. Exosomal miRNAs are encapsulated within extracellular vesicles, ensuring their stability, while ctDNA fragments are shed freely into circulation. Once in the bloodstream, these biomarkers can be isolated from blood samples and analyzed using advanced molecular techniques such as quantitative PCR (qPCR), next-generation sequencing, and digital PCR (dPCR). Unlike PSA, which merely indicates biochemical changes, exosomal miRNAs and ctDNA provide deeper insights into tumor burden, recurrence risk, and molecular alterations, making them valuable tools for surveillance.

### 3.2. Exosomal miRNAs versus circulating tumor DNA: a comparative analysis

While both exosomal miRNAs and ctDNA offer potential advantages, their comparative diagnostic accuracy for early recurrence detection remains unclear. Exosomal miRNAs may provide higher specificity due to their tumor-derived origin and stability, whereas ctDNA offers real-time insights into tumor evolution and resistance mechanisms^[10]^ (Table 1). Some studies suggest that exosomal miRNAs outperform ctDNA in early recurrence detection, while others highlight ctDNA’s superiority in tracking genomic alterations.^[11]^ Given the complementary nature of these biomarkers, integrating both approaches may enhance diagnostic accuracy and provide a more comprehensive assessment of recurrence risk.^[12]^ However, further research is needed to determine the optimal biomarker panel and establish standardized protocols for clinical application.

**Table 1:** Conceptual comparison of exosomal miRNAs and circulating tumor DNA (ctDNA) in prostate cancer recurrence detection.

Feature	Exosomal miRNAs	Circulating tumor DNA (ctDNA)	Clinical implications
Biological source	Encapsulated in extracellular vesicles released by tumor cells	Free-floating DNA fragments shed from tumor cells	Determines stability and detection feasibility
Detection techniques	qPCR, microarrays, RNA sequencing (NGS-based approaches)	Digital PCR, next-generation sequencing (NGS), methylation profiling	Advanced technologies needed for clinical adoption
Stability in circulation	High (protected within vesicles, resistant to RNAses)	Moderate (prone to degradation, affected by clearance rate)	Affects reliability for early detection
Tumor specificity	High (derived from tumor microenvironment)	Moderate (includes background ctDNA from non-tumor sources)	Determines accuracy in distinguishing recurrence
Sensitivity for early recurrence	High (detects molecular changes before PSA rise)	Variable (depends on tumor shedding and ctDNA levels)	Affects clinical timing of intervention
Genomic insights	Functional regulatory roles in tumor progression, therapy resistance	Direct detection of tumor mutations, epigenetic alterations	Helps guide targeted therapy strategies
Limitations	Expression variability across patients, lack of standardized protocols	Low ctDNA levels in early recurrence, high cost of sequencing	Standardization and validation required for clinical implementation
Potential for integration	Can complement ctDNA and PSA for enhanced diagnostic accuracy	Valuable for tracking tumor evolution and resistance mechanisms	A multi-biomarker approach may improve surveillance

qPCR = quantitative PCR, PSA = prostate-specific antigen.

Exosomal miRNAs are small non-coding RNAs enclosed within vesicles that play a crucial role in intercellular communication and gene regulation. Their vesicular protection ensures greater stability in circulation, preventing degradation and allowing for reliable detection even at low concentrations.^[13]^ Several specific miRNAs, such as miR-141, miR-375, and miR-21, have been associated with prostate cancer progression and recurrence, making them strong candidates for biomarker development.^[14]^

In contrast, ctDNA consists of fragmented genetic material released into the bloodstream through tumor cell apoptosis and necrosis. Unlike exosomal miRNAs, ctDNA is not protected by vesicles, making it more prone to degradation.^[15]^ However, ctDNA offers a crucial advantage in providing real-time genomic insights into tumor evolution, mutation profiling, and treatment resistance.^[16]^ By analyzing ctDNA, clinicians can detect genetic alterations, such as mutations and methylation changes, that may indicate aggressive disease progression or potential therapeutic targets.^[17]^

**Figure 2.**
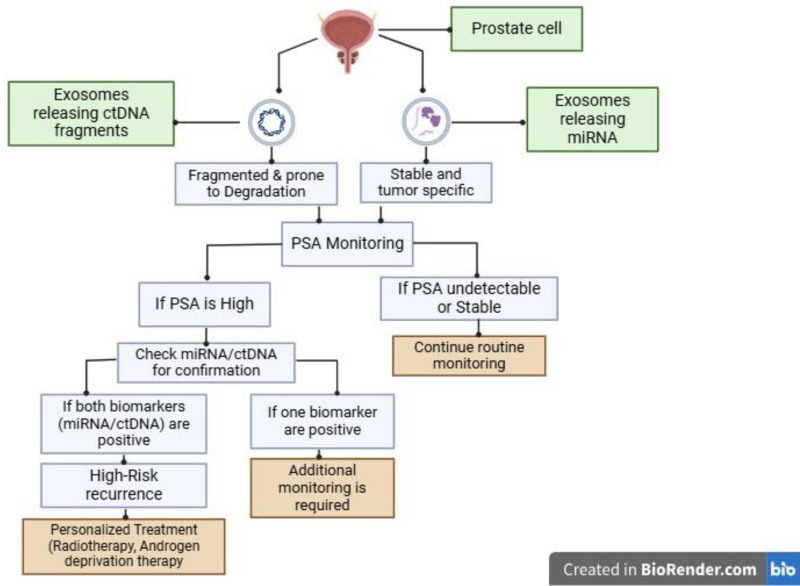
Mechanism of exosomal miRNA and ctDNA release and multi-biomarker approach. ctDNA = circulating tumor DNA, miRNAs = exosomal microRNAs.

As depicted in Figure 2, exosomal miRNAs are generally considered more stable and tumor-specific, whereas ctDNA allows for mutation tracking and dynamic assessment of tumor burden. While exosomal miRNAs may provide a higher specificity in identifying prostate cancer recurrence, ctDNA serves as a valuable tool for monitoring clonal evolution and treatment response. Given their distinct advantages, neither biomarker can fully replace the other, highlighting the need for an integrative approach to recurrence detection.

The study evaluated the risk of immune-related adverse events (irAEs) in patients with advanced gastrointestinal cancer treated with immune checkpoint inhibitors (ICIs) versus chemotherapy, using data from the U.S. TriNetX database (2015–2022). ICIs were associated with a significantly higher incidence of irAEs compared to chemotherapy alone. CTLA-4 inhibitors showed a higher irAE rate than PD-1/PD-L1 inhibitors. Notably, patients who developed irAEs had lower mortality after propensity score matching (HR 0.661; 95% CI: 0.620–0.704; *P* < .01). These real-world findings help inform the benefit-risk profile of ICIs in gastrointestinal cancer treatment.^[18]^

### 3.3. A multi-biomarker approach for enhanced recurrence detection

Considering the strengths and limitations of each biomarker, an integrative approach combining PSA, exosomal miRNAs, and ctDNA may offer the most accurate and personalized strategy for post-radical prostatectomy surveillance.^[19]^ Exosomal miRNAs provide high specificity and biological stability, potentially reducing the false positives frequently encountered with PSA alone.^[20]^ Concurrently, ctDNA offers insights into tumor dynamics, including mutational profiles and emerging resistance mechanisms, which can inform treatment selection and prognostication.^[21]^

However, current studies evaluating these biomarkers exhibit substantial heterogeneity in patient populations, sample processing techniques, and detection platforms, making it difficult to draw definitive comparisons or establish standardized thresholds. Variability in assay sensitivity and specificity further complicates clinical interpretation. Moreover, the lack of consensus on optimal sampling time points and biomarker panels limits reproducibility across studies. These methodological inconsistencies contribute to the variable diagnostic performance reported in the literature.

Cost-effectiveness and logistical feasibility also remain significant barriers. High-throughput sequencing and exosome isolation technologies, while promising, may be inaccessible in routine clinical settings due to cost, technical complexity, and infrastructure demands. Additionally, regulatory approval and clinical validation in large, prospective cohorts are necessary before widespread adoption.

To support broader clinical engagement, a visual comparison summarizing the advantages, limitations, and clinical applications of PSA, exosomal miRNAs, and ctDNA may enhance accessibility and facilitate informed decision-making among practitioners.

### 3.4. Challenges and future directions

Despite their promise, several challenges hinder the clinical translation of exosomal miRNAs and ctDNA in post-radical prostatectomy surveillance. Standardization of sample collection, processing, and data analysis is essential to ensure reproducibility across studies.^[22]^ Additionally, cost and accessibility remain barriers to widespread adoption, as liquid biopsy technologies require advanced sequencing and bioinformatics expertise^[23]^ (Table 2). Future research should focus on large, multi-center prospective studies to validate biomarker performance and explore their integration with other modalities such as imaging and artificial intelligence-based predictive models.^[24,25]^ Ultimately, a multi-biomarker approach combining exosomal miRNAs, ctDNA, and traditional PSA monitoring may offer the most effective strategy for early recurrence detection, guiding personalized treatment decisions and improving patient outcomes.^[26]^

**Table 2:** Challenges and future directions for liquid biopsy biomarkers in prostate cancer recurrence detection.

Challenge	Impact on exosomal miRNAs	Impact on ctDNA	Future considerations
Standardization of detection	Lack of consensus on isolation and analysis methods	Variability in sequencing depth and detection cutoffs	Establishing universal protocols for clinical use
Sensitivity and specificity	High specificity but variability in expression across patients	Sensitive but affected by tumor shedding dynamics	Optimizing detection algorithms to improve reliability
Cost and accessibility	Requires specialized RNA sequencing techniques	NGS and digital PCR remain costly and resource-intensive	Cost-effective assays needed for widespread adoption
Validation in large cohorts	Small-scale studies with inconsistent results	Needs more prospective trials with long-term follow-up	Large, multi-center studies essential for clinical translation
Clinical utility	Promising for early recurrence detection, but requires further validation	Potentially useful for guiding targeted therapies and real-time monitoring	Integration with PSA and imaging may enhance decision-making

ctDNA = circulating tumor DNA, miRNAs = exosomal microRNAs, NGS = next-generation sequencing, PSA = prostate-specific antigen.

This narrative review is limited by potential selection bias, variability in study designs, and the lack of standardized protocols for exosomal miRNAs and ctDNA assessment. Many studies are still in early stages, with small sample sizes and retrospective designs, limiting definitive conclusions. Additionally, the rapid evolution of liquid biopsy research necessitates further large-scale, prospective studies to validate these biomarkers and establish standardized clinical guidelines for prostate cancer recurrence detection.

## 4. Conclusion

Exosomal miRNAs and ctDNA have emerged as promising liquid biopsy biomarkers for the early detection of prostate cancer recurrence after radical prostatectomy.^[27]^ Exosomal miRNAs offer stability and tumor specificity, while ctDNA provides real-time insights into tumor genomics and clonal evolution.^[28]^ Although both biomarkers show potential advantages over PSA alone, their comparative diagnostic accuracy remains unclear due to variability in study methodologies and the lack of standardized protocols. Given their complementary nature, a multi-biomarker approach integrating exosomal miRNAs, ctDNA, and traditional PSA monitoring may enhance recurrence detection and guide personalized treatment strategies.^[29]^ However, further large-scale, prospective studies are needed to validate their clinical utility, refine detection techniques, and establish standardized guidelines for their integration into routine clinical practice.^[30]^

## Author contributions

**Conceptualization:** Hamza Bashir, Muhammad Waqas Afzal, Asad Mehmood, Maham Afzal, Muhammad Wasi Hassan, Abida Parveen.

**Data curation:** Maham Afzal, Muhammad Wasi Hassan.

**Investigation:** Hamza Bashir.

**Project administration:** Abida Parveen.

**Resources:** Hamza Bashir, Maham Afzal, Abida Parveen.

**Software:** Muhammad Waqas Afzal.

**Validation:** Hamza Bashir, Muhammad Wasi Hassan.

**Visualization:** Muhammad Waqas Afzal, Asad Mehmood, Maham Afzal, Muhammad Wasi Hassan, Abida Parveen.

**Writing – original draft:** Muhammad Waqas Afzal, Asad Mehmood.

**Writing – review & editing:** Hamza Bashir, Muhammad Waqas Afzal, Asad Mehmood, Maham Afzal, Muhammad Wasi Hassan, Abida Parveen.
